# Poly(I:C) Challenge Alters Brain Expression of Oligodendroglia-Related Genes of Adult Progeny in a Mouse Model of Maternal Immune Activation

**DOI:** 10.3389/fnmol.2020.00115

**Published:** 2020-06-30

**Authors:** Xiao-Fan Zhang, Ting Chen, Aifen Yan, Jia Xiao, Yong-Li Xie, Jing Yuan, Pin Chen, Anderson On-Lam Wong, Yang Zhang, Nai-Kei Wong

**Affiliations:** ^1^Department of Psychiatry, Tongji Hospital, Tongji Medical College, Huazhong University of Science and Technology, Wuhan, China; ^2^Department of Psychiatry, The University of Hong Kong, Hong Kong, China; ^3^CAS Key Laboratory of Tropical Marine Bio-resources and Ecology (LMB), Key Laboratory of Applied Marine Biology of Guangdong Province and Chinese Academy of Sciences (LAMB), South China Sea Institute of Oceanology, Chinese Academy of Sciences, Guangzhou, China; ^4^School of Stomatology and Medicine, Foshan University, Foshan, China; ^5^School of Biomedical Sciences, Li Ka Shing Faculty of Medicine, The University of Hong Kong, Hong Kong, China; ^6^National Clinical Research Center for Infectious Diseases, Shenzhen Third People’s Hospital, The Second Hospital Affiliated to Southern University of Science and Technology, Shenzhen, China; ^7^School of Biological Sciences, The University of Hong Kong, Hong Kong, China

**Keywords:** maternal immune activation (MIA), poly(I:C), *SOX10*, myelin-associated glycoprotein (*MAG*), transferrin (*Tf*), schizophrenia, autistic spectrum disorder, white matter

## Abstract

**Background**: Altered white matter connectivity, as evidenced by pervasive microstructural changes in myelination and axonal integrity in neuroimaging studies, has been implicated in the development of autism spectrum disorder (ASD) and related neurodevelopmental conditions such as schizophrenia. Despite an increasing appreciation that such white matter disconnectivity is linked to social behavior deficits, virtually no etiologically meaningful myelin-related genes have been identified in oligodendrocytes, the key myelinating cells in the CNS, to furnish an account on the causes. The impact of neurodevelopmental perturbations during pregnancy such as maternal immune activation (MIA) on these genes in memory-related neural networks has not been experimentally scrutinized.

**Methods**: In this study, a mouse model of MIA by the viral dsRNA analog poly(I:C) was employed to mimic the effects of inflammation during pregnancy. Transcriptional expression levels of selected myelin- or oligodendroglia-related genes implicated in schizophrenia or ASD development were analyzed by *in situ* hybridization (ISH) and quantitative real-time PCR (qRT-PCR) with brain samples from MIA and control groups. The analysis focused on *SOX-10* (SRY-related HMG-box 10), *MAG* (myelin-associated glycoprotein), and *Tf* (transferrin) expression in the hippocampus and the surrounding memory-related cortical regions in either hemisphere.

**Results**: Specifically, ISH reveals that in the brain of prenatal poly(I:C)-exposed mouse offspring in the MIA model (gestation day 9), mRNA expression of the genes *SOX10*, *MAG* and *Tf* were generally reduced in the limbic system including the hippocampus, retrosplenial cortex and parahippocampal gyrus on either side of the hemispheres. qRT-PCR further confirms the reduction of *SOX10*, *MAG*, and *Tf* expression in the medial prefrontal cortex, sensory cortex, amygdala, and hippocampus.

**Conclusions**: Our present results provide direct evidence that prenatal exposure to poly(I:C) elicits profound and long-term changes in transcript level and spatial distribution of myelin-related genes in multiple neocortical and limbic regions, notably the hippocampus and its surrounding memory-related neural networks. Our work demonstrates the potential utility of oligodendroglia-related genes as biomarkers for modeling neurodevelopmental disorders, in agreement with the hypothesis that MIA during pregnancy could lead to compromised white matter connectivity in ASD.

## Background

Growing evidence suggests that functional disconnectivity within and across large-scale neural networks is a central hallmark of clinically overlapping neurodevelopmental disorders such as autism spectrum disorder (ASD) and schizophrenia (Sharma et al., 2017; van den Heuvel et al., 2017). Indeed, numerous studies by diffusion tensor imaging (DTI; Cheung et al., 2008; Nickel et al., 2017; Boets et al., 2018; d’Albis et al., 2018) and histopathology (Drakesmith et al., 2016; Aoki et al., 2017) have demonstrated regional deficits in the white matter volume, microstructural alterations in white matter tracts, and aberrant oligodendroglial features in patients with schizophrenia or ASD. Post-mortem microarray and quantitative real-time PCR (qRT-PCR) analysis have revealed a reduction in the expression of oligodendroglia-related genes in multiple brain regions of schizophrenic patients (Tkachev et al., 2003; Aberg et al., 2006; Sokolov, 2007) and children with ASD (Broek et al., 2014; Tian et al., 2017). In particular, altered expression patterns of myelin- or oligodendroglia-related genes reported in schizophrenia patients include the oligodendrocyte markers SRY-related HMG-box 10 (*SOX10*, Iwamoto et al., 2005; Jones et al., 2007; Maeno et al., 2007; Kato and Iwamoto, 2014), myelin-associated glycoprotein (*MAG*, Fujita et al., 1998; Yin et al., 1998; Felsky et al., 2012) and transferrin (Escobar Cabrera et al., 1994; Guardia Clausi et al., 2010), which are critically involved in myelination and oligodendrocyte progenitor differentiation (Baumann and Pham-Dinh, 2001). Previously, both clinical and experimental studies have proposed a variety of potential diagnostic or prognostic biomarkers for ASD based on biological samples from peripheral tissues or body fluids such as serum and urine (Bridgemohan et al., 2019; Salloum-Asfar et al., 2019; Swanson and Hazlett, 2019; Shen et al., 2020). In contrast, information on the clinical profiles of myelin- or oligodendroglia-related genes in patients with ASD is virtually wanting, necessitating an urgent need to address this important gap. It is being increasingly appreciated that dysfunction of oligodendrocytes contributes at least in part to abnormal synaptic communication and uncoupled white matter connection reported in schizophrenia (Olney et al., 1999; McGlashan and Hoffman, 2000; Najjar and Pearlman, 2015; Vikhreva et al., 2016; Yuan et al., 2016) and ASD studies (Zoghbi and Bear, 2012; Hahamy et al., 2015). However, the causal mechanisms of oligodendroglial abnormality have remained poorly understood and particularly difficult to clarify in human subjects. Also, there has been no consensus as to which of the molecular markers should be profiled toward a systematic understanding of oligodendroglial abnormality in animal models for ASD or schizophrenia, including the MIA (maternal immune activation) models (Estes and McAllister, 2016).

Modeling MIA by polyriboinosinic-polyribocytidylic acid [poly(I:C)] induction in animals is a robust method for investigating neurodevelopmental disorders (Careaga et al., 2017; Mueller et al., 2018). In this study, we employed a poly(I:C)-induced MIA model in mice to study any changes in the oligodendroglia-related genes of interest in the brain of adult progeny. Specifically, we exposed pregnant mice to poly(I:C), a synthetic mimetic of viral dsRNA analog, a well-documented mouse model of MIA. We and others have reported that poly(I:C)-induced MIA precipitates altered brain and behavioral phenotypes in offspring that mirror abnormalities observed in ASD and related neurodevelopmental conditions such as schizophrenia (Meyer et al., 2006; Li et al., 2015; Wei et al., 2016; Wu et al., 2018).

Another objective of this study was to identify which of the myelin-rich neural substrates are potentially affected by inflammation during pregnancy concerning the expression of the oligodendroglia-related genes. To this end, we performed *in situ* hybridization to directly assess the effects of MIA on the transcriptional expression of the oligodendroglia-related genes including *SOX10*, *MAG*, and transferrin (*Tf*) in the brain of adult mouse offspring. Based on the literature on white matter disconnectivity associated with the hippocampus-amygdala complex or temporal lobes in schizophrenia and ASD (Sigmundsson et al., 2001; Herbert et al., 2003; Ha et al., 2015; Sforazzini et al., 2016; van den Heuvel et al., 2016), we anticipated that a loss of oligodendroglia-related gene markers would be detected in the corresponding brain regions in prenatal MIA-exposed mice. Also, based on initial histological observations in our *in situ* hybridization analysis that bilateral hemispheric differences may exist for *SOX10*, *MAG*, and *Tf* among certain sub-cortical regions of the mouse brain, we employed an experimental design in quantitative real-time PCR that permits left-right brain comparison of the genes. This design helps to validate *in situ* hybridization observations, and complement knowledge gained from whole-brain approaches to the study of white matter changes in MIA models.

## Materials and Methods

### Animals

Breeding and mating of female and male C57BL6/N mice were performed in compliance with the guidelines of the Laboratory Animal Unit (LAU), the University of Hong Kong. Timed-pregnant mice were kept in a 12:12-h reversed light-dark cycle (light onset at 19:00), and temperature and humidity-controlled (21 ± 1°C, 55 ± 5%) animal vivarium. Animals had access ad libitum to food and water supplied by the LAU. All experiments had been approved by the Committee on the Use of Live Animals in Teaching and Research at the University of Hong Kong, and every effort had been made to minimize the number of animals used and their suffering.

### Prenatal Treatment

Prenatal treatment was performed as previously reported by our group (Li et al., 2015). Briefly, poly(I:C; potassium salt, Sigma Aldrich) with a dosage of 5 mg/kg in an injection volume of 5 ml/kg was freshly prepared on the day of injection and administered to pregnant dams on GD9 (gestation day 9) *via* the tail vein under mild physical constraint. Control animals were similarly administered with 5 ml/kg saline *via* tail vein on GD9. The animals were returned to their home cages after injection. The resulting offspring from six litters [poly(I:C) *n* = 3; control *n* = 3] were weaned and sexed at postnatal day (PND) 21. Pups were weighed and littermates of the same sex were caged separately (3–4 per cage). Only adult male offspring were used in subsequent experiments.

### Molecular Cloning

Murine brain tissues were collected from stock adult mice, which were then immediately frozen in liquid nitrogen and stored at −80°C until use for total RNA isolation. Total RNA was extracted from mouse brains by using TRIzol (Invitrogen, San Diego, CA, USA) and reverse-transcribed with SuperScript II (Invitrogen). Based on the cDNAs from reverse transcription, gene-specific primers for *SOX10*, *L-MAG* (long-form *MAG*), and *Tf* (Supplementary Table S1) were used to clone the coding sequences (CDS) of the oligodendroglia-related genes. The PCR products were purified by using a BigDye Sequencing Kit (Applied Biosystems, Forster City, CA, USA). The purified cDNAs were then subcloned into a pGEM-T Easy cloning vector (Promega, Madison, WI, USA), and subjected to DNA sequencing for confirmation of the identity of oligodendroglia-related genes. The resulting cDNA plasmids were used in subsequent experiments for DIG-labeled probe synthesis.

### DIG-Labeled Probe Synthesis

Double-strand (ds) DIG (digoxygenin)-labeled DNA probes were prepared with a PCR DIG Probe Synthesis Kit (Roche) by using specific PCR primers (Supplementary Table S2) according to the instructions of the manufacturer. Labeled probes were analyzed by gel electrophoresis to check for “size shift” after DIG-labeling. The performance of labeled probes in terms of hybridization selectivity and titering of probes used in hybridization reaction was confirmed in spot tests. Storage of DIG-labeled probe stock solutions was done in small aliquots (2–4 μl volume) at –80°C for future use.

### Tissue Sectioning and *in situ* Hybridization

*In situ* hybridization was performed on several selected regions of the mouse brain, based on methods previously reported by our group (Jiang et al., 2008). Brains (*n* = 3 per group) from adult offspring of different pregnant mice (*n* = 3 per group) either exposed to poly(I:C)- or saline at GD9 were collected. The brains were perfused under terminal anesthesia, fixed in 4% paraformaldehyde, and embedded in paraffin wax. Brain sections of 5 μm in thickness were prepared on a Leica RM2135 microtome (Leica Microsystems, Wetzlar, Germany) and mounted onto pre-coated Superfrost Plus glass slides (Thermo Scientific, Waltham, MA, United States). The tissue sections were stored at −80°C for subsequent experiments. For *in situ* hybridization, brain sections were dewaxed with xylene, rehydrated with decreasing levels of ethanol, and post-fixed briefly in 4% paraformaldehyde. After that, the sections were digested with proteinase K (Roche), washed, and incubated at 37°C for 10 min with hybridization solution [4× saline sodium citrate (SSC), 1× Denhardt’s solution, 10% dextran sulfate, 10 mM DTT, 40% formamide, 1 mg/ml salmon sperm DNA, 500 μg/ml yeast tRNA, 5 μg/ml poly deoxy adenylic acid, and 100 μg/ml polyadenylic acid]. For *in situ* hybridization, DIG-labeled DNA probes for mouse *SOX10, L-MAG*, and *Tf* were added to brain sections, followed by incubation at 42°C overnight in a humidified chamber. On the following day, post-hybridization washing was performed in decreasing concentrations of SSC solution at 42°C. Signal development was conducted with an anti-DIG antibody (1:500, Roche) by using NBT and BCIP as the substrates. In these experiments, hybridization with coronal sections of the saline-challenged mouse brain was used as a control.

**Table 1:** Primers and amplification conditions for quantitative PCR in this study.

	PCR conditions							
Gene target/Accession no. (Primer sequences, 5′-3′)	Denaturing	Annealing	Extension	Detection	Cycle no.	Tm	Amplif. efficiency	Product size
*SOX10***/NM_011437.1**	94°C	60°C	72°C	87°C
AACGGTGCCAGCAAGAGCAA	30 s	30 s	30 s	20 s	35	91°C	1.98	245 bp
CGAGGTTGGTACTTGTAGTC								
*Transferrin***/AF440692.1**	94°C	60°C	72°C	87°C
GGCCTGACTCCGAACAACCTGAAGC	30 s	30 s	30 s	20 s	35	91°C	1.98	260 bp
CTGCCCGAGAAGAAACTGGACACAG								
*L-MAG***/NM_010758**	94°C	62°C	72°C	87°C
AGTGAGAAGCAGCGCCTGGGATCTG	30 s	30 s	30 s	20 s	35	88°C	1.86	168 bp
TCACTTGACTCGGATTTCTGCATACTC								
*T-MAG***/NM_010758**	94°C	50°C	72°C	87°C
GATGCCCTCGACCATCTCAGCCTTC	30 s	30 s	30 s	20 s	35	91°C	1.76	278 bp
GGAAATAGTATTTGCCTCCCAGCTC								
*GAPDH***/M32599.1**	94°C	62°C	72°C	85°C
GTGGAGCCAAACGGGTCATCATCT	30 s	30 s	30 s	20 s	30	89°C	1.84	188 bp
GGTCATGAGCCCTTCCACAAT

### RNA Preparation and Gene Expression Studies

Total RNA from individual mouse (*n* = 3 per group) brain regions including mPFC, hippocampus, amygdala, neocortex, and thalamus from an independent batch of the samples used by *in situ* experiments, was isolated with TRIzol reagent (Invitrogen). Next, 4 μg of each total RNA sample was digested with DNase I (Invitrogen) and reverse-transcribed into cDNA (RT) with SuperScript II (Invitrogen). Gene-specific primers for real-time PCR were designed based on the sequences of mouse *SOX10, L-MAG, T-MAG* (total *MAG*), *Tf*, and GAPDH. The RT samples obtained were then subjected to quantitative PCR in a RotorGene 6,000 Real-time PCR System (Corbett Research, Eight Mile Plains, New South Wales, Australia) and PCR reactions were conducted with a Light-cycler DNA Master SYBR Green I Kit (Roche). The primer sequences and real-time PCR reactions for gene expression studies are as shown in Table 1.

### Data Transformation and Statistical Analysis

For quantitative real-time PCR for transcript expression of *SOX10, L-MAG, T-MAG*, and *Tf*, standard curves were constructed with serial dilutions of plasmids carrying the ORF of the respective gene targets, as described previously (Wong et al., 2013). After linear regression of threshold cycle values, standard curves within the dynamic range and with a correlation coefficient of ≥0.95 were used for data calibration. The raw data for the respective gene targets were quantified in terms of femtomole transcript detected per microgram of total RNA. There was no significant difference for *GAPDH* mRNA expression in our experiments, thus *SOX10, L-MAG, T-MAG*, and *Tf* mRNA expression were normalized as a ratio of *GAPDH* mRNA detected in the same sample for statistical analysis. Data expressed as mean ± SEM. were analyzed by using Student’s *t*-test; significance was determined at *p < 0.05, **p < 0.01, or ***p < 0.001 by using SPSS (IBM Software).

## Results

### *In situ* Hybridization

*SOX10, L-MAG*, and *Tf* transcript expression was generally reduced in several brain regions (both right and left sides) including the hippocampus, retrosplenial granular cortex (RSG), and retrosplenial dysgranular cortex (RSD), in the prenatal poly(I:C)-exposed group relative to saline-exposed control.

**Figure 1:**
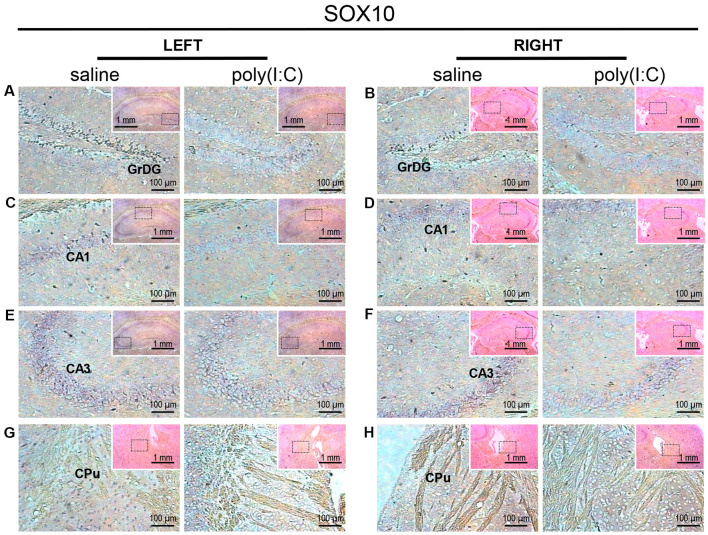
*In situ* hybridization with a SRY-related HMG-box 10 (*SOX10*)-specific probe in the left and right hippocampi and striatums of adult mouse offspring. *SOX10* was detected as dark blue puncta. Four ROIs (regions of interest) in the brain of poly(I:C)- or saline-exposed mice were examined, namely, granular layer of the dentate gyrus (GrDG; panels **A,B**), CA1 (panels **C,D**), CA3 panels **(E,F)** and CPu (caudate-putamen; panels **G,H**) in a coronal section. Results are representative of two independent experiments. Insets show the approximate location of the ROIs in each panel relative to a larger area of the tissue. Representative images in detail were acquired at 100× magnification, and those in the inset (top right of each detailed image) at 25× magnification.

Within the hippocampus, the granular layer of the dentate gyrus (GrDG) showed the highest density of *SOX10* signals, while the CA1 and CA3 regions showed mild to moderate expression in saline control mice (Figure 1). Besides, *SOX10* signals were also observed in the left caudate-putamen (CPu; Figure 1G), but no *SOX10* signals were detected on the right side of CPu (Figure 1H) in saline control mice. In the neocortex of saline-exposed control, *SOX10* signals were abundantly detected in the medial part of the RSD and RSG (Figure 4A), the somatosensory cortex (SC), auditory cortex (AC) and the piriform cortex (PC; Supplementary Figure S1). However, in the prenatal poly(I:C)-exposed group, *SOX10* signals were either very weak in the hippocampus (GrDG, CA1, and CA3), left CPu, RSG and RSD, or SC, AC or PC (Figures 1, 4A and Supplementary Figure S1) compared to that of the saline control mice or virtually undetectable in the right CPu, which was similar to the case of the control group (Figure 1H).

**Figure 2:**
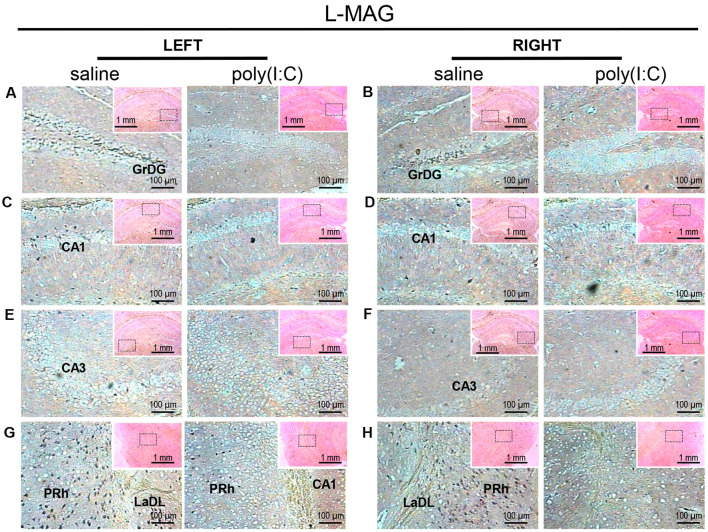
*In situ* hybridization with an long-form myelin-associated glycoprotein (*L-MAG*)-specific probe in the left and right hippocampi and parahippocampal regions of adult mouse offspring. *L-MAG* was detected as dark blue puncta. Five ROIs in the brain of poly(I:C)- or saline-exposed mice were examined, namely, granular layer of the dentate gyrus (GrDG; panels **A,B**), CA1 (panels **C,D**), CA3 panels **(E,F)**, perirhinal cortex (PRh) and lateral amygdaloid nucleus dorsolateral part (LaDL; panels **G,H**) in a coronal section. Results are representative of two independent experiments. Insets show the approximate location of the ROIs in each panel relative to a larger area of the tissue. Representative images in detail were acquired at 100× magnification, and those in the inset (top right of each detailed image) at 25× magnification.

**Figure 3:**
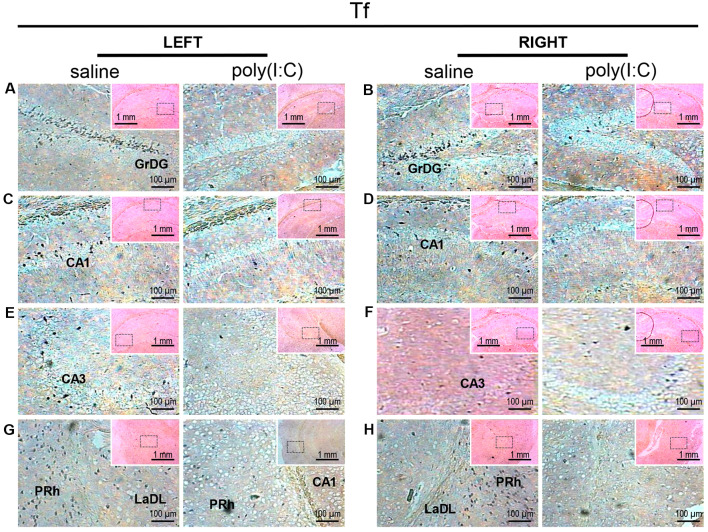
*In situ* hybridization with a transferrin (*Tf*)-specific probe in the left and right hippocampi and parahippocampal regions of adult mouse offspring. *Tf* was detected as dark blue puncta. Five ROIs in the brain of poly(I:C)- or saline-exposed mice were examined, namely, granular layer of the dentate gyrus (GrDG; panels **A,B**), CA1 (panels **C,D**), CA3 panels **(E,F)**, perirhinal cortex (PRh) and lateral amygdaloid nucleus dorsolateral part (LaDL; panels **G,H**) in a coronal section. Results are representative of two independent experiments. Insets show the approximate location of the ROIs in each panel relative to a larger area of the tissue. Representative images in detail were acquired at 100× magnification, and those in the inset (top right of each detailed image) at 25× magnification.

**Figure 4:**
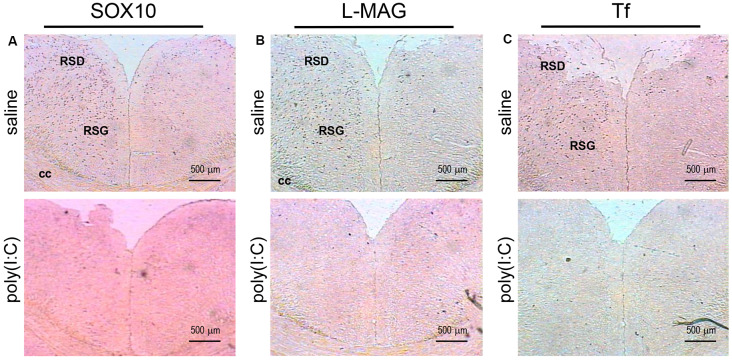
*In situ* hybridization with a *SOX10*-, *L-MAG*- or *Tf*-specific probe in the medial part of the retrosplenial cortex of adult mouse offspring. *SOX10* mRNA was detected as dark blue puncta in panel **(A)**, *L-MAG* mRNA in panel **(B)**, and *Tf* mRNA in panel **(C)**. Two ROIs in the brain of poly(I:C)- or saline-exposed mice were examined, namely, the retrosplenial dysgranular cortex (RSD) and retrosplenial granular cortex (RSG) in a coronal section. Results are representative of two independent experiments. cc, corpus callosum. Representative images were acquired at 40× magnification.

**Figure 5:**
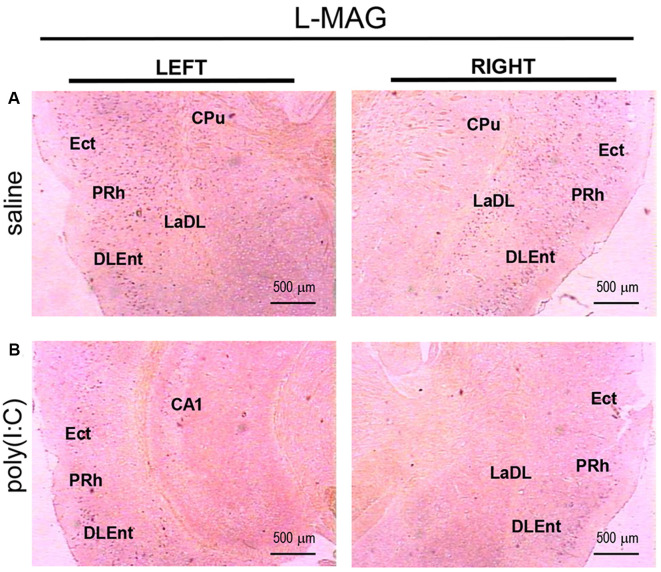
*In situ* hybridization with an *L-MAG*-specific probe in the left and right parahippocampal regions of adult mouse offspring. *L-MAG* mRNA was detected as dark blue puncta in panel **(A)** for prenatal saline-exposed control, and in panel **(B)** for prenatal poly(I:C)-exposed group. Several ROIs in the brain of poly(I:C)- or saline-exposed mice were examined, namely, the ectorhinal cortex (Ect), perirhinal cortex (PRh), and dorsolateral entorhinal cortex (DLEnt) in a coronal section. Results are representative of two independent experiments. CPu, caudate putamen; LaDL, lateral amygdaloid nucleus dorsolateral part. Representative images were acquired at 25× magnification.

For *L-MAG* expression in saline-exposed control, the hippocampal regions (i.e., GrDG, CA1, CA3; Figure 2) again showed the strongest signals in terms of intensity and density compared to poly(I:C)-challenged mice. *L-MAG* signals were abundantly detected in an area encompassing the perirhinal cortex (PRh) and the lateral amygdaloid nucleus dorsolateral part (LaDL; Figures 2G,H) in either hemisphere of the saline-exposed control. When viewed as a larger anatomical neighborhood, the ectorhinal cortex (Ect), PRh, and dorsolateral entorhinal (DLEnt) in the left brain seemed to have marginally stronger *L-MAG* signals than their counterparts in the right brain (Figure 5). Also, *L-MAG* expressing cells were abundantly found in the medial retrosplenial cortex (RSD and RSG; Figure 4B; Supplementary Figures S2A,B). In contrast, *L-MAG* mRNA expression of the prenatal poly(I:C)-exposed group was conspicuously absent or dramatically reduced in the same brain regions examined above when compared with that of the saline-exposed control (Figures 2, 4, 5).

Transcript expression patterns of *Tf* strikingly resembled that of *SOX10* and *L-MAG* in the hippocampus (Figure 3) of saline-exposed control. Again, the left GrGD showed the highest density and intensity of *Tf* signals among the hippocampal ROIs examined. In both the left and right parahippocampal regions, moderately strong *Tf* signals could be found (Figures 3G,H). *Tf* signals were also abundantly detected in the retrosplenial cortex (Figure 4C) and various sensory cortical regions (Supplementary Figure S3). In contrast, in the prenatal poly(I:C)-exposed mouse brain, there was a prevalent and remarkable reduction in *Tf* signals in the same brain regions examined above (Figures 3, 4, Supplementary Figure S3).

Lastly, in the thalamus (Th), *SOX10, L-MAG* and *Tf* signals were undetectable in both the prenatal poly(I:C)- and saline-exposed groups (Supplementary Figures S4A, S5A, S6A), whereas in the ventral hypothalamus (VH), *SOX10, L-MAG*, and *Tf* ignals were moderate in the saline-exposed control, but again undetectable in the prenatal poly(I:C)-exposed group (Supplementary Figures S4B, S5B, S6B).

### qRT-PCR Analysis on *SOX10*, *MAG*, and *Tf* mRNA Expression

To clarify whether poly(I:C) induced MIA would give rise to nonspecific modulatory effects on global gene expression in the mouse brain, we included the housekeeping gene *GAPDH* as a reference gene. Also, the qRT-PCR results reveal that there was no significant difference between the prenatal poly(I:C)- and saline-exposed groups in all the brain regions compared (Figure 9), which is consistent with the oligodendroglia-related genes results for *in situ* hybridization for these regions (Supplementary Figures S1–S6).

**Figure 6:**
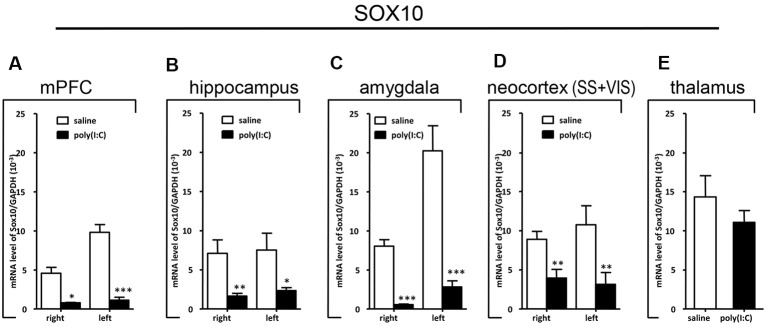
Quantitative real-time polymerase chain reaction (qRT-PCR) evaluation of effects of poly(I:C)-induced maternal immune activation (MIA) on *SOX10* mRNA expression in selected brain regions (panels **A–E**: mPFC, hippocampus, amygdala, neocortex (SS+VIS) and thalamus). Tissues from the left and right hemispheres of adult mouse offspring [*n* = 3 for prenatal poly(I:C) or saline-exposed groups] were analyzed. Each bar represents mean ± SEM. Significance was determined at *p < 0.05, **p < 0.01, and ***p < 0.001 between treatments. SS + VIS: sensory and visual cortices; mPFC: the medial prefrontal cortex.

**Figure 7:**
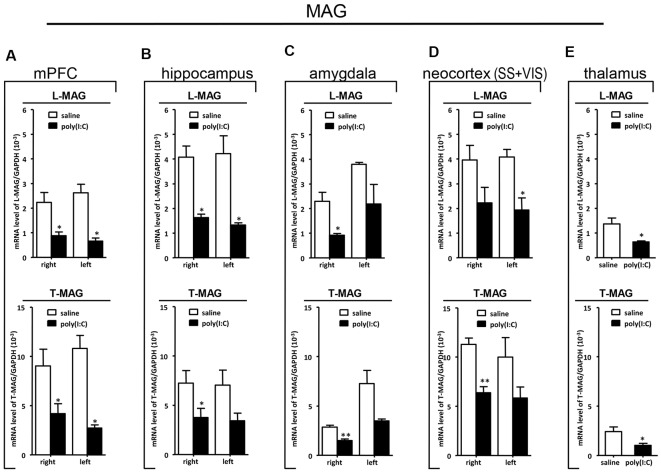
qRT-PCR evaluation of effects of poly(I:C)-induced MIA on *L-MAG* and *T-MAG* mRNA expression in selected brain regions (panels **A–E**: mPFC, hippocampus, amygdala, neocortex (SS+VIS) and thalamus). Tissues from the left and right hemispheres of adult mouse offspring [*n* = 3 for prenatal poly(I:C) or saline-exposed groups] were analyzed. Each bar represents mean ± SEM. Significance was determined at *p < 0.05 and **p < 0.01 between treatments. SS + VIS: sensory and visual cortices; mPFC: the medial prefrontal cortex.

**Figure 8:**
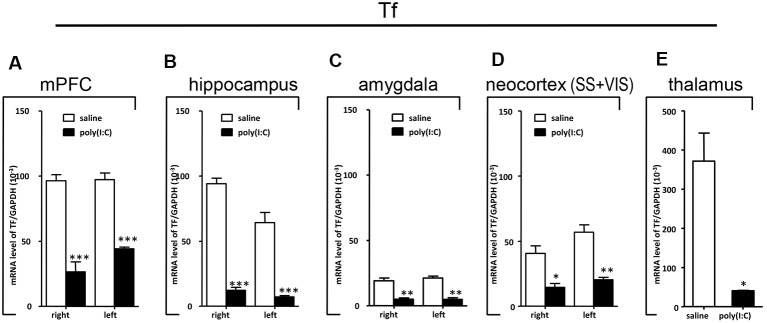
qRT-PCR evaluation of effects of poly(I:C)-induced MIA on *Tf* mRNA expression in selected brain regions (panels **A–E**: mPFC, hippocampus, amygdala, neocortex (SS+VIS) and thalamus). Tissues from the left and right hemispheres of adult mouse offspring (*n* = 3 for prenatal poly(I:C) or saline-exposed groups) were analyzed. Each bar represents mean ± SEM. Significance was determined at *p < 0.05, **p < 0.01, and ***p < 0.001 between treatments. SS + VIS: sensory and visual cortices; mPFC: the medial prefrontal cortex.

**Figure 9:**
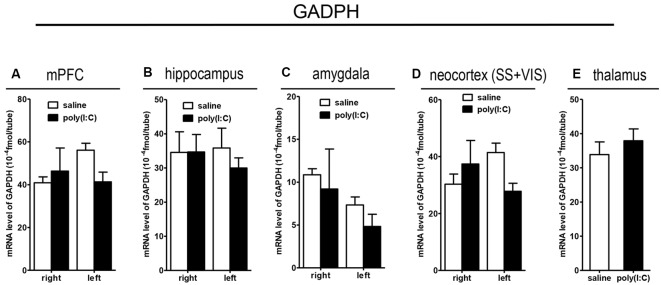
qRT-PCR evaluation of effects of poly(I:C)-induced MIA on *GAPDH* mRNA expression in selected brain regions (panels **A–E**: mPFC, hippocampus, amygdala, neocortex (SS+VIS) and thalamus). Tissues from the left and right hemispheres of adult mouse offspring [*n* = 3 for prenatal poly(I:C) or saline-exposed groups] were analyzed. Each bar represents mean ± SEM. SS + VIS: sensory and visual cortices; mPFC: the medial prefrontal cortex.

In the mPFC, hippocampus, amygdala, and parts of the neocortex in both hemispheres, the transcript levels of *SOX10* in prenatal poly(I:C)-exposed group were significantly lower (by at least one-fold difference) than that of saline-exposed control (Figure 6). In contrast, in the thalamus, there was a marginal but nonsignificant difference in *SOX10* mRNA expression between prenatal poly(I:C)- and saline-exposed groups (Figure 6E). In both right and left mPFC, mRNA expression of *L-MAG* and *T-MAG* in prenatal poly(I:C)-exposed group was significantly lower (*p* < 0.05; Figure 7A). In the hippocampus, prenatal poly(I:C) challenge induced marked reduction in *L-MAG* mRNA expression in either hemisphere, while a reduction in *T-MAG* expression was found only on the right side (*p* < 0.05; Figure 7B). In the amygdala, poly(I:C)-exposed group showed decreased expression of *L-MAG* and *T-MAG* on the right but not the left side (Figure 7C). In the neocortex of poly(I:C)-exposed mice, significantly lower *T-MAG* mRNA expression appeared on the right side, while decreased *L-MAG* mRNA expression appeared on the left side (Figure 7D). Additionally, in the thalamus, poly(I:C) treatment induced a moderate but significant effect in lowering mRNA levels of *L-MAG* and *T-MAG* (Figure 7E). Consistent with *in situ* hybridization results, prevalent and marked reduction of *Tf* mRNA was found in all brain regions examined in either hemisphere of prenatal poly(I:C)-exposed mice (Figure 8). Among these, the mPFC, amygdala, and neocortex showed about two-fold decrease of *Tf* signals (Figures 8A,C,D), while the hippocampus and thalamus showed at least four-fold difference (Figures 8B,E).

## Discussion

In this study, a remarkable reduction of *SOX10, MAG*, and *Tf* transcript expression were seen in nearly all neocortical and limbic structures in prenatal poly(I:C)-exposed mice. Specifically, we found that several brain regions with prominent functions in learning, memory and social interaction (including the hippocampus, amygdaloid nuclei, entorhinal cortex, and retrosplenial cortex) showed markedly reduced *SOX10, MAG*, and *Tf* mRNA expression, suggesting that they may be high-risk regions susceptible to the effects of poly(I:C)-induced MIA during pregnancy. This disruption of the normal course of development could result in a compromised capacity to maintain synchrony of widely distributed neural networks, which is ultimately manifested in the heterogeneity of symptoms and cognitive deficits of schizophrenia and autism (Bartzokis, 2002; Uhlhaas and Singer, 2010; French et al., 2015; Zalesky et al., 2015).

The transcriptional deficits of oligodendroglia-related genes in the different brain regions that we have found in this study are in agreement with the anatomical substrates reported in structural MRI studies (Bauman and Kemper, 1985; Aylward et al., 1999; Rojas et al., 2004; Honea et al., 2005; Nacewicz et al., 2006; Ellison-Wright and Bullmore, 2009). Abnormalities of the fronto-temporoparietal cortex network, hippocampus, and cerebellum and white matter connecting these regions seem to underscore the pathology of ASD and, that such changes could result from abnormal brain development during early life (Brambilla et al., 2003; Barnea-Goraly et al., 2004).

Myelin deficits shared by the hippocampal formation and the entorhinal cortex support the idea that white matter disconnectivity affects interconnected components of neural networks, rather than isolated brain loci. Within the hippocampus, the GrDG consistently showed the greatest intensity and density of *SOX10, L-MAG*, and *Tf* signals in saline-exposed control (Figures 1–3). Accordingly, the qualitative differences observed between the poly(I:C)- and saline-exposed groups were also greatest in this subregion of the hippocampus. The entorhinal cortex (layer II) is physically adjacent to the hippocampal formation and provides primary projections to both the dentate gyrus and CA3 (Tamminga et al., 2010). The dentate gyrus has been deemed a gateway structure receiving and transmitting neural inputs into the hippocampus. It is interesting to note that the apparent reduction in oligodendroglia-related gene expression in the GrDG of prenatal poly(I:C)-exposed brain (Figures 1A,B, 2A,B, 3A,B) parallels the well-documented reduction in dentate gyrus glutamatergic output (Tamminga et al., 2010), an abnormality traditionally attributed to neuronal dysfunction.

Apart from the hippocampus and entorhinal cortex, a memory-related brain region that showed clear transcriptional alterations was the retrosplenial cortex (RSD and RSG; Figure 4). The retrosplenial cortex is known to be reciprocally interconnected with both the hippocampus and the prefrontal cortex and is involved in a range of cognitive functions including episodic memory, spatial working memory, and even emotion (Maddock, 1999; Vann et al., 2009). Indeed, schizophrenic and ASD patients have been shown to have major deficits in spatial working memory, previously thought to stem from prefrontal cortical dysfunction (Park and Holzman, 1992; Demetriou et al., 2018). For *SOX10, L-MAG*, and *Tf* in our study, transcript expression patterns in the RSD/RSG showed a discernible interhemispheric difference, with signals being preferentially stronger on the left brain of saline-exposed control. Due to the generally very low *SOX10, L-MAG*, and *Tf* signals being detected in prenatal poly(I:C)-exposed brain, this interhemispheric difference was not observed in *in situ* hybridization. In qRT-PCR, *SOX10, L-MAG/T-MAG*, and *Tf* transcript levels were reduced by over three folds (Figure 6A), one fold (Figure 7A), and two folds (Figure 8A), respectively, in the mPFC, an observation consistent with hypoactivity of executive-control in schizophrenia. Likewise, there was a clear trend of reduction in *SOX10, L-MAG/T-MAG*, and *Tf* transcript levels in the amygdala (Figures 6C, 7C, 8C), a center that modifies plasticity of fear memories and projects into the hippocampus and other limbic structures. Of note, *SOX10* mRNA levels were reduced by close to three folds in the right and left amygdala in mice prenatal exposed to poly(I:C), whereas only about one-fold difference was observed for *L-MAG/T-MAG* mRNA in the right but not left amygdala. As a structural protein with cell signaling functions mediated by its additional cytoplasmic domain, *L-MAG* contributes to most functions of total *T-MAG* in the CNS. The proportionally more significant reduction in *SOX10* mRNA expression could mean that *SOX10* as a transcription factor contributes significant weight to altered myelination or oligodendrocyte function in the postnatal offspring.

**Figure 10:**
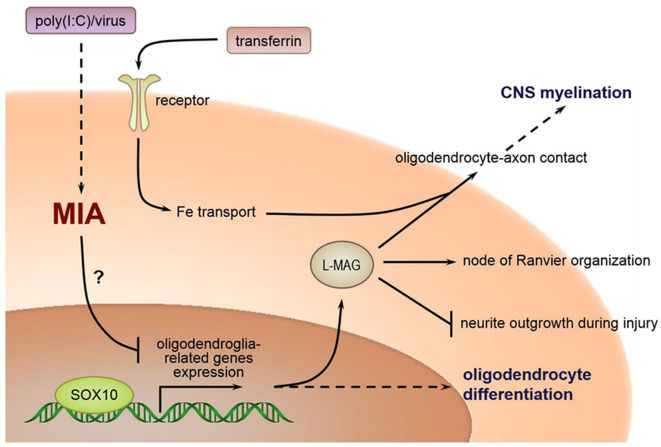
Relations between MIA during pregnancy and selected oligodendroglia-related genes involved in myelination.

The relationship between poly(I:C) exposure induced MIA during pregnancy and selected oligodendroglia-related genes involved in myelination are as delineated in Figure 10. As posited in previous research, in humans, the temporal extent of brain plasticity can extend into the middle age, when maximal white matter volume and myelination are reached in frontal lobes and association areas (Bartzokis, 2002; Insel, 2010). However, the expression of certain genes critical to myelination is linearly driven and activatable only at early developmental stages. For example, *SOX10* expression is possible in embryonic neurons but becomes exclusively restricted to oligodendrocytes in postnatal life. Disruption of normal gene expression patterns (especially *SOX10*, which interacts with other transactivators, e.g., Olig1, Olig2, etc.) early in brain development could well have a more profound effect than the case of later stage disturbances. Consistent with our findings, reduced *SOX10* expression was found in the hippocampus and anterior cingulate cortex (ACC) of subjects with schizophrenia (Dracheva et al., 2006). Also, *SOX10* is a key regulator of oligodendrocyte differentiation by directly controlling the transcription of genes implicated in this process (Stolt et al., 2002). *Tf* acts as trophic and survival factors for neurons and astrocytes, with important implications for oligodendrocyte functions (Baumann and Pham-Dinh, 2001; Figure 10). *MAG* is selectively located in periaxonal Schwann cells and oligodendroglia membranes of myelin sheaths. It promotes the differentiation, maintenance, and survival of oligodendrocytes (Quarles, 2007). Also, *MAG* helps structure nodes of Ranvier and inhibits neurite regeneration after injury (Schnaar and Lopez, 2009; Figure 10). Decreased transcript expression of *Tf* and *MAG* in the white matter of the ACC has been reported in subjects with schizophrenia (McCullumsmith et al., 2007). Furthermore, expression of these oligodendrocyte-related genes is disrupted in ASD-related animal models. For example, Fatemi et al. (2009) demonstrated that middle second (E16) and late second (E18) trimester infections of pregnant mice with human influenza virus reduced the expression of myelin basic protein (*MBP*) and *MAG* in the cerebella of mouse offspring (Fatemi et al., 2009), and compromised their white matter integrity as measured by diffusion tensor MRI, respectively (Fatemi et al., 2008). To our best knowledge, our present study presents the first evidence that prenatal poly(I:C) challenge precipitates profoundly reduced transcriptional expression of the oligodendroglia-related genes including *SOX10, MAG* and *Tf* in memory-related neural networks in either brain hemisphere of the adult mouse offspring. These molecular changes may underscore the white matter alterations reported in MIA models elsewhere. It is tempting to suggest identification of these molecular markers may provide opportunities for better understanding ASD pathogenesis in the clinical and experimental settings, for which further interrogation is warranted.

The prevalent nature of gene expression changes seen in many brain regions has unexpectedly stretched the scope of this study. As a result, we only chose to focus on describing the effects of poly(I:C)-induced MIA on a few ROIs known to be involved in learning, memory, and social interaction. For *in situ* hybridization, we examined a typical coronal section (Bregma –1.70 mm) revealing the hippocampus but did not explore some other brain regions deemed important in canonical schizophrenia or autism pathophysiology such as the prefrontal cortex. Similarly, a sagittal section would reveal more information on oligodendroglial markers in anatomical structures relevant to autism, such as the cerebellum. In qRT-PCR, we chose to examine transcript expression patterns separately in the left and right brains, but our findings were restricted by relatively small sample sizes. Differences in tissue sampling methods used in *in situ* hybridization and qRT-PCR mean that their results are coupled to differential spatial resolution and variability. Therefore, qualitative differences seen in histology (e.g., subregions of the hippocampus) may not always be reflected in quantitative differences in qRT-PCR (e.g., whole hippocampus).

Overall, our work is relatively exploratory. It attempted to address the need for specific molecular biomarkers relevant to myelin- or oligodendroglia-related changes in murine MIA models, in particular, that of poly(I:C)-induced MIA in pregnant mice. While previously some of our works supported the utility of C57BL/6N mice in evaluating behavior on social-affective functions (Zhang et al., 2015a,b), we did not include a behavioral assessment component in the current study. For future investigations involving interventional measures, more in-depth behavioral and histological characterization in conjunction with quantitation of new molecular markers related to myelination changes could be an interesting approach.

## Conclusion

In the current study, prenatal poly(I:C) exposure dramatically altered the spatial distribution and transcript level of oligodendroglial molecular signatures including *SOX10, MAG*, and *Tf* in memory and social interaction-related brain regions including mPFC, hippocampus, amygdala, neocortex, and thalamus, which lends support to the notion that white matter abnormalities have their anatomical substrates at the molecular level in neurodevelopmental disorders such as ASD and schizophrenia. Given that myelination and oligodendroglial function are coupled to synaptic communication, altered expression patterns of oligodendroglia-related genes seem a useful molecular indicator for evaluating myelination status or oligodendroglial function concerning certain cognitive deficits found in schizophrenia or ASD patients. The extensive spatial scope of neural substrates showing altered oligodendroglia-related gene expression agrees with the observation that schizophrenia and ASD are heterogeneous disorders where inter-gray matter miscommunication and white matter disconnectivity co-occur. We believe that further study of prophylactic medicine at the perinatal or postnatal stages to ameliorate or rescue oligodendrocyte dysfunction is warranted, as it may open up new avenues for the prevention of neurodevelopmental or psychiatric disorders.

## Data Availability Statement

The datasets analyzed in this article are not publicly available. Requests to access the datasets should be directed to wongnksz@163.com.

## Ethics Statement

The experiments had been reviewed and approved by the Committee on the Use of Live Animals in Teaching and Research at the University of Hong Kong, and every effort had been made to minimize the number of animals used and their suffering.

## Author Contributions

N-KW, YZ, and AW conceived and designed the experiments. X-FZ, TC, AY, PC, and Y-LX performed the experiments. N-KW, Y-LX, JY, and JX analyzed the imaging data. X-FZ, YZ, and TC performed the statistical analysis. N-KW, X-FZ, YZ, and TC wrote the article. All authors read and approved the final manuscript.

## Conflict of Interest

The authors declare that the research was conducted in the absence of any commercial or financial relationships that could be construed as a potential conflict of interest.

## Supplementary Material

FIGURE S1 *In situ* hybridization with a *SOX10*-specific probe in the left and right cortical regions of adult mouse offspring. *SOX10* mRNA was detected as dark blue puncta. Several ROIs in the brain of poly(I:C)- or saline-exposed mice were examined, namely, retrosplenial granular cortex (RSG) and retrosplenial dysgranular cortex (RSD; panels **A,B**), the somatosensory cortex (SC; panels **C,D**), auditory cortex (AC; panels **E,F**), and the piriform cortex (PC; panels **G,H**). Results are representative of two independent experiments. cc: corpus callosum. Representative images were acquired at 25× magnification.

FIGURE S2 *In situ* hybridization with an *L-MAG*-specific probe in the left and right cortical regions of adult mouse offspring. *L-MAG* mRNA was detected as dark blue puncta. Several ROIs in the brain of poly(I:C)- or saline-exposed mice were examined, namely, retrosplenial granular cortex (RSG) and retrosplenial dysgranular cortex (RSD; panels **A,B**), the somatosensory cortex (SC; panels **C,D**), auditory cortex (AC; panels **E,F**), and the piriform cortex (PC; panels **G,H**). Results are representative of two independent experiments. cc: corpus callosum. Representative images were acquired at 25× magnification.

FIGURE S3 *In situ* hybridization with a *Tf*-specific probe in the left and right cortical regions of adult mouse offspring. *Tf* mRNA was detected as dark blue puncta. Several ROIs in the brain of poly(I:C)- or saline-exposed mice were examined, namely, retrosplenial granular cortex (RSG) and retrosplenial dysgranular cortex (RSD; panels **A,B**), the somatosensory cortex (SC; panels **C,D**), auditory cortex (AC; panels **E,F**), and the piriform cortex (PC; panels **G,H**). Results are representative of two independent experiments. cc: corpus callosum. Representative images were acquired at 25× magnification.

FIGURE S4 *In situ* hybridization with a *SOX10*-specific probe in the thalamus and ventral hypothalamus of adult mouse offspring. *SOX10* mRNA was detected as dark blue puncta. Several ROIs in the brain of poly(I:C)- or saline-exposed mice were examined, namely, thalamus (Th; panel **A**), and ventral hypothalamus (VH; panel **B**). Results are representative of two independent experiments. Representative images were acquired at 25× magnification.

FIGURE S5 *In situ* hybridization with an *L-MAG*-specific probe in the thalamus and ventral hypothalamus of adult mouse offspring. *L-MAG* mRNA was detected as dark blue puncta. Several ROIs in the brain of poly(I:C)- or saline-exposed mice were examined, namely, thalamus (Th; panel **A**), and ventral hypothalamus (VH; panel **B**). Results are representative of two independent experiments. Representative images were acquired at 25× magnification.

FIGURE S6 *In situ* hybridization with a *Tf*-specific probe in the thalamus and ventral hypothalamus of adult mouse offspring. *Tf* mRNA was detected as dark blue puncta. Several ROIs in the brain of poly(I:C)- or saline-exposed mice were examined, namely, thalamus (Th; panel **A**), and ventral hypothalamus (VH; panel **B**). Results are representative of two independent experiments. Representative images were acquired at 25× magnification.

TABLE S1 Primers for generating cDNA templates in molecular cloning.

TABLE S2 Primers for gene-specific DIG-labeled probes used in in situ hybridization.

## Abbreviations

ASD: autism spectrum disorder
MIA: maternal immune activation
poly(I:C): polyriboinosinic- polyribocytidylic acid
ISH: *in situ* hybridization (ISH)
qRT-PCR: quantitative real-time polymerase chain reaction
*MAG*: myelin associated glycoprotein
*L-MAG*: long-form *MAG*
*SOX-10*: SRY-related HMG-box 10
*Tf*: transferrin
GD9: gestation day 9
CDS: coding sequences
RSG: retrosplenial granular cortex
RSD: retrosplenial dysgranular cortex
GrDG: granular layer of dentate gyrus
CPu: caudate putamen
SC: somatosensory cortex
AC: auditory cortex
PC: piriform cortex
pRh: perirhinal cortex.
