# Supplementary figures and images for: Poly(I:C) Challenge Alters Brain Expression of Oligodendroglia-Related Genes of Adult Progeny in a Mouse Model of Maternal Immune Activation
Source: Front Mol Neurosci. 2020 Jun 30;13:115. doi: 10.3389/fnmol.2020.00115 (PMC7340146; doi:10.3389/fnmol.2020.00115)

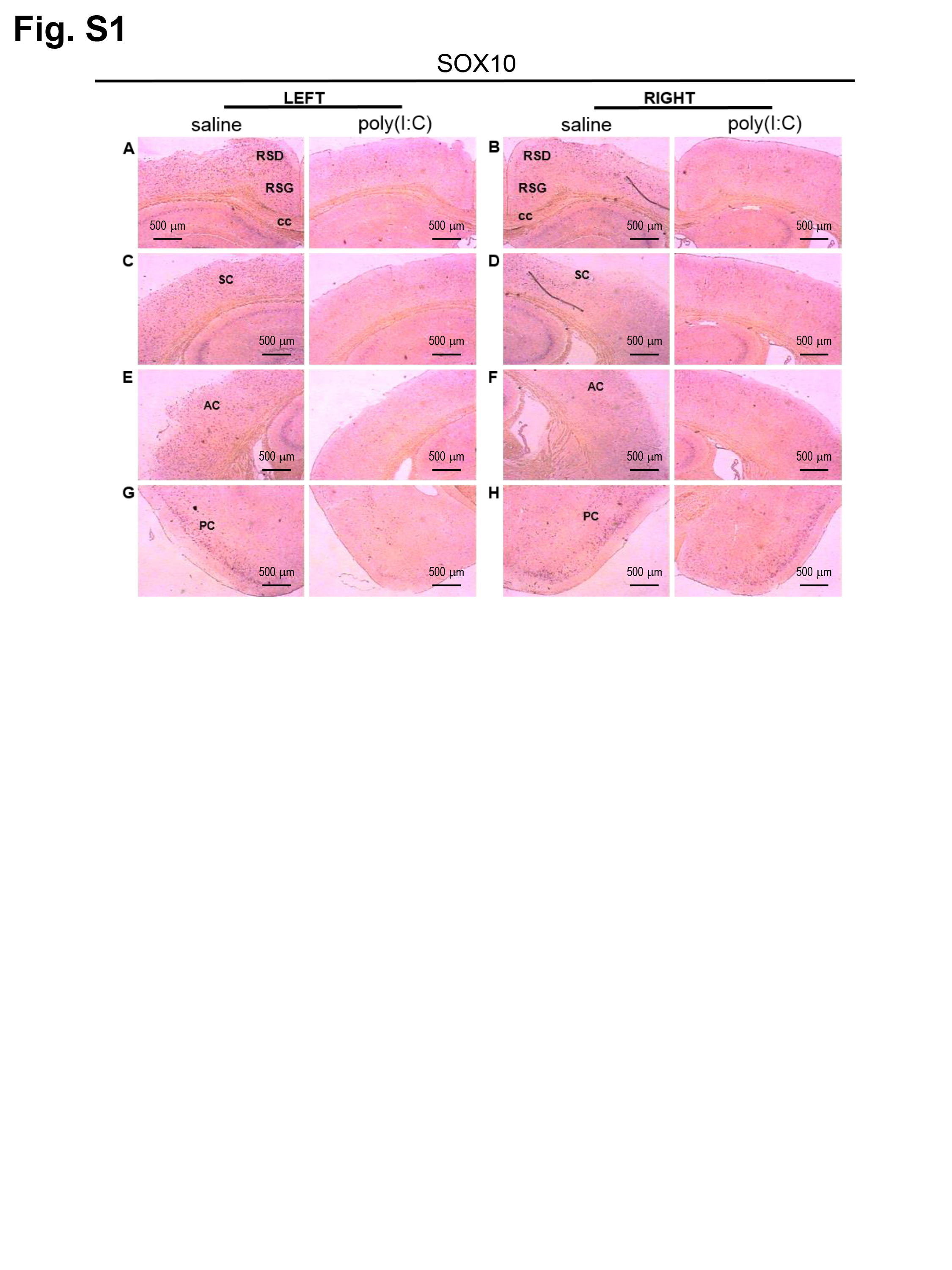

Supplement: FIGURE S1 — In situ hybridization with a SOX10-specific probe in the left and right cortical regions of adult mouse offspring. SOX10 mRNA was detected as dark blue puncta. Several ROIs in the brain of poly(I:C)- or saline-exposed mice were examined, namely, retrosplenial granular cortex (RSG) and retrosplenial dysgranular cortex (RSD; panels A,B), the somatosensory cortex (SC; panels C,D), auditory cortex (AC; panels E,F), and the piriform cortex (PC; panels G,H). Results are representative of two independent experiments. cc: corpus callosum. Representative images were acquired at 25× magnification. [file Image_1.JPEG]

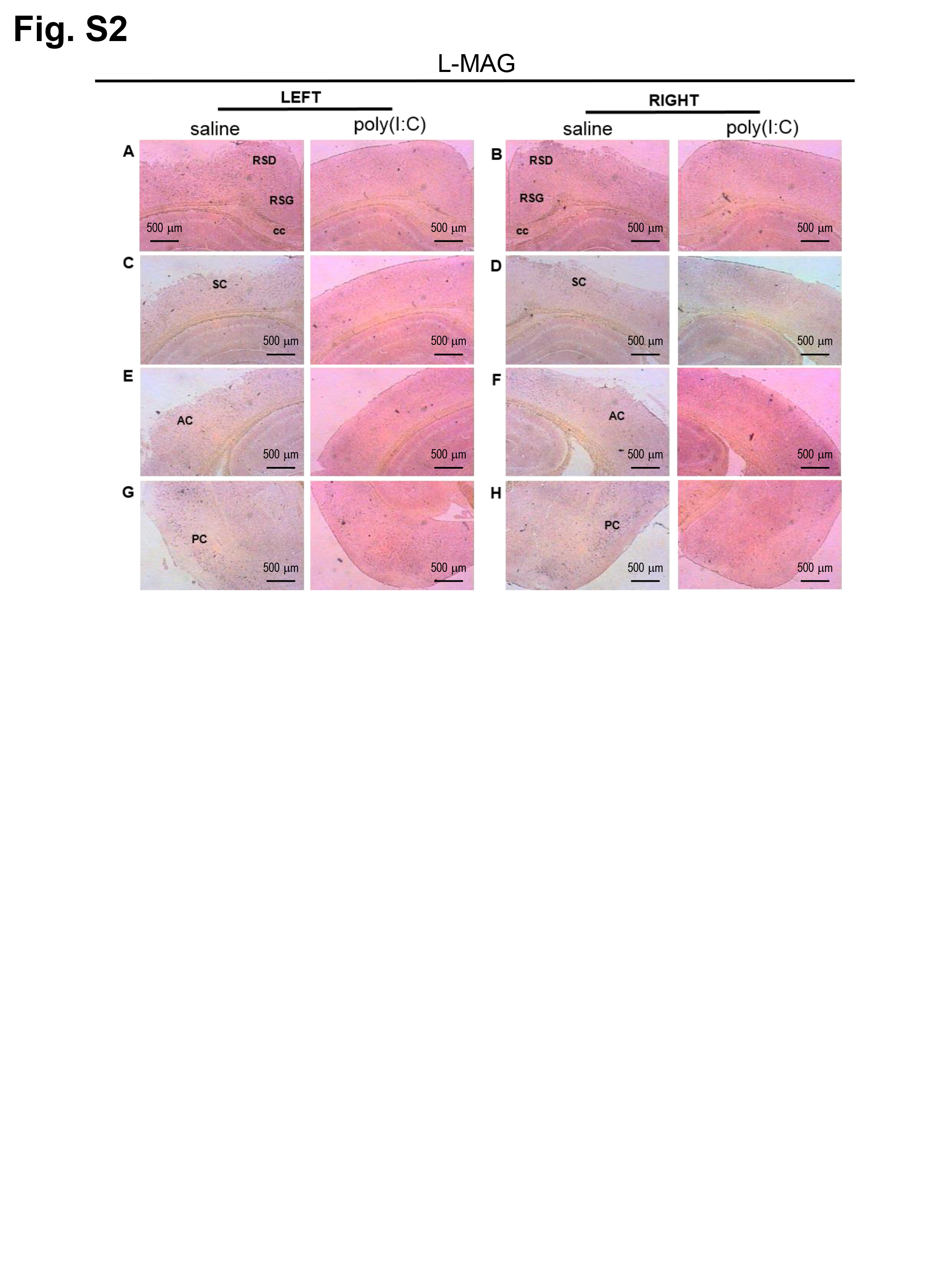

Supplement: FIGURE S2 — In situ hybridization with an L-MAG-specific probe in the left and right cortical regions of adult mouse offspring. L-MAG mRNA was detected as dark blue puncta. Several ROIs in the brain of poly(I:C)- or saline-exposed mice were examined, namely, retrosplenial granular cortex (RSG) and retrosplenial dysgranular cortex (RSD; panels A,B), the somatosensory cortex (SC; panels C,D), auditory cortex (AC; panels E,F), and the piriform cortex (PC; panels G,H). Results are representative of two independent experiments. cc: corpus callosum. Representative images were acquired at 25× magnification. [file Image_2.JPEG]

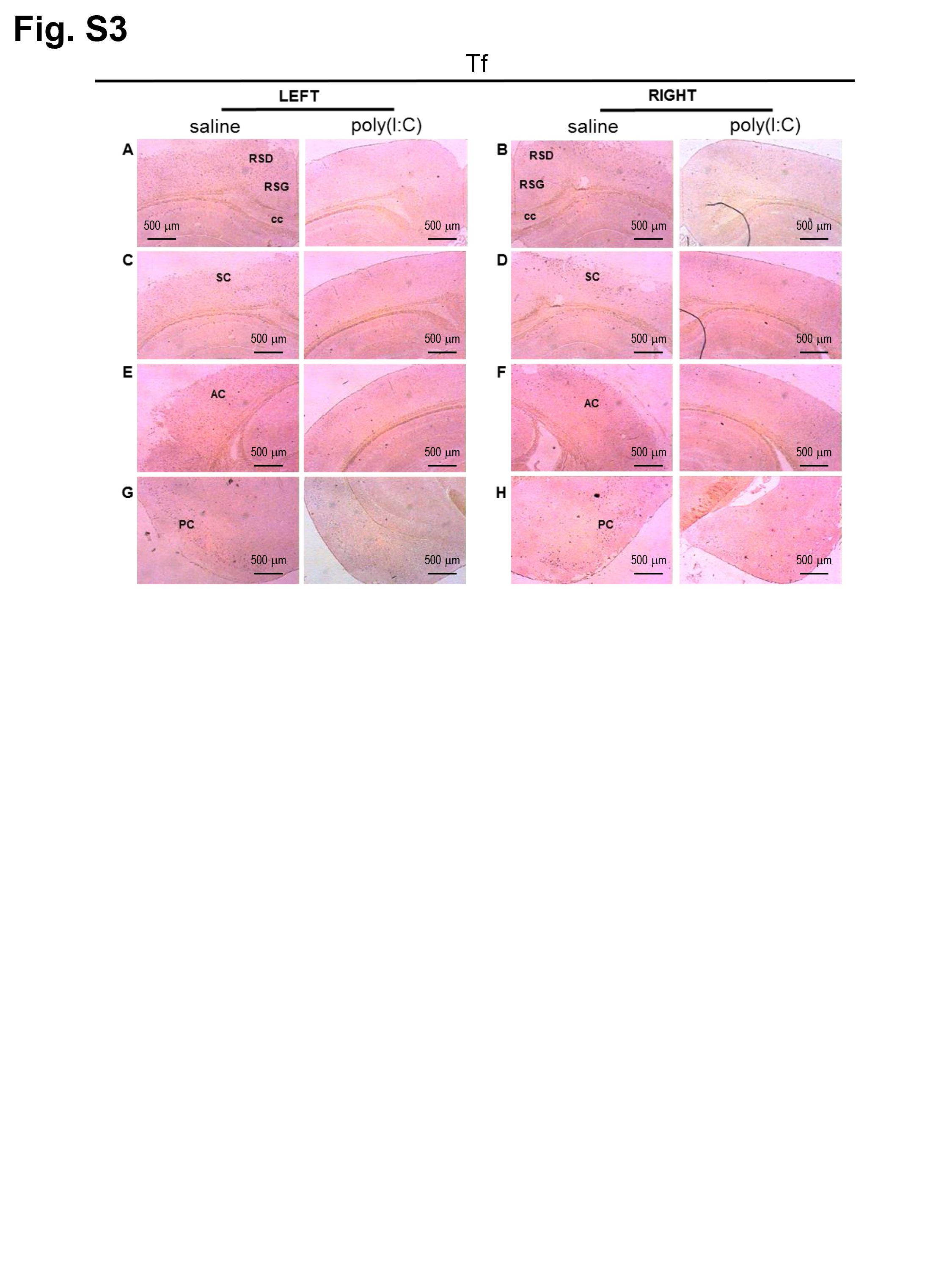

Supplement: FIGURE S3 — In situ hybridization with a Tf-specific probe in the left and right cortical regions of adult mouse offspring. Tf mRNA was detected as dark blue puncta. Several ROIs in the brain of poly(I:C)- or saline-exposed mice were examined, namely, retrosplenial granular cortex (RSG) and retrosplenial dysgranular cortex (RSD; panels A,B), the somatosensory cortex (SC; panels C,D), auditory cortex (AC; panels E,F), and the piriform cortex (PC; panels G,H). Results are representative of two independent experiments. cc: corpus callosum. Representative images were acquired at 25× magnification. [file Image_3.JPEG]

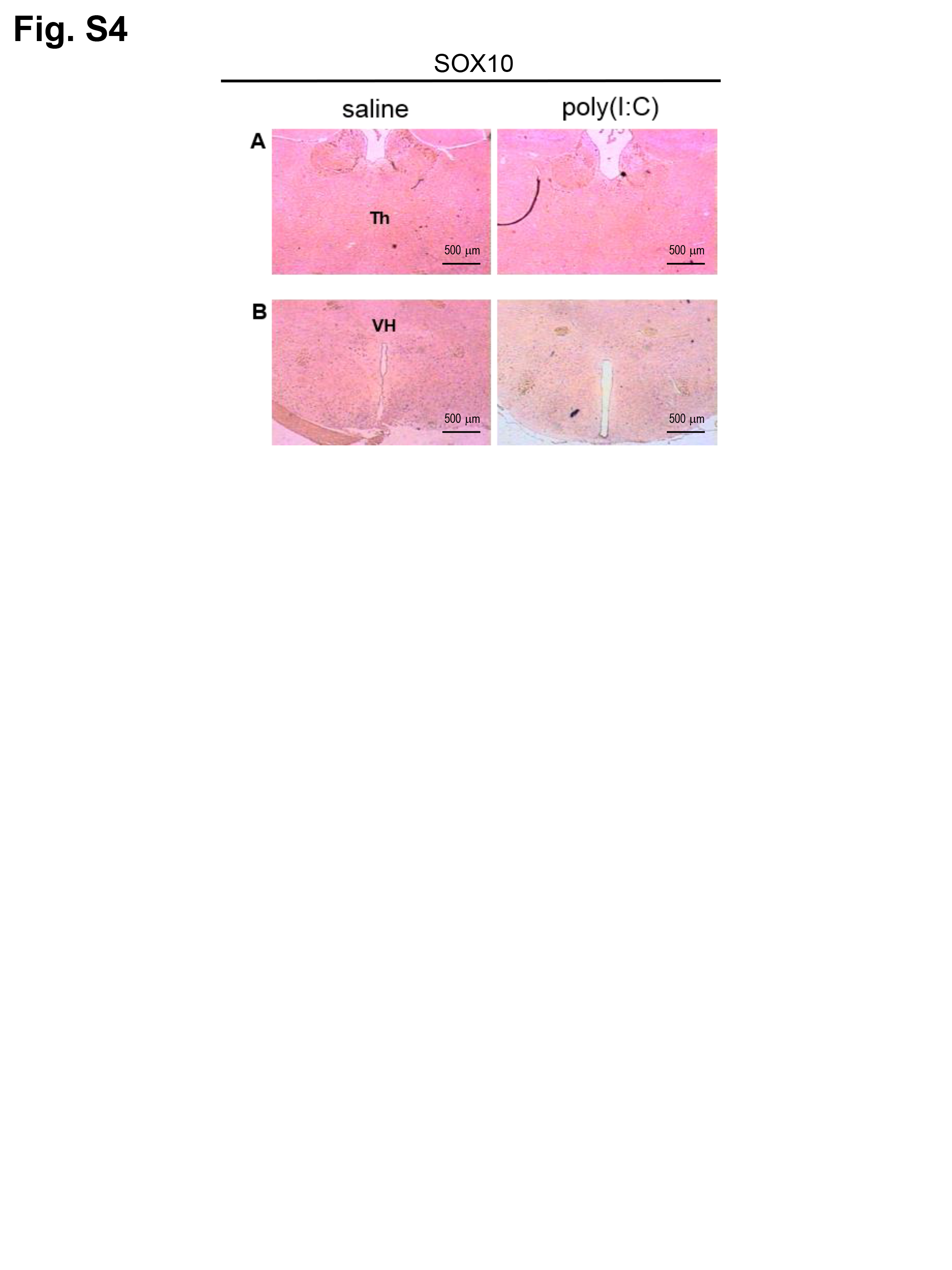

Supplement: FIGURE S4 — In situ hybridization with a SOX10-specific probe in the thalamus and ventral hypothalamus of adult mouse offspring. SOX10 mRNA was detected as dark blue puncta. Several ROIs in the brain of poly(I:C)- or saline-exposed mice were examined, namely, thalamus (Th; panel A), and ventral hypothalamus (VH; panel B). Results are representative of two independent experiments. Representative images were acquired at 25× magnification. [file Image_4.JPEG]

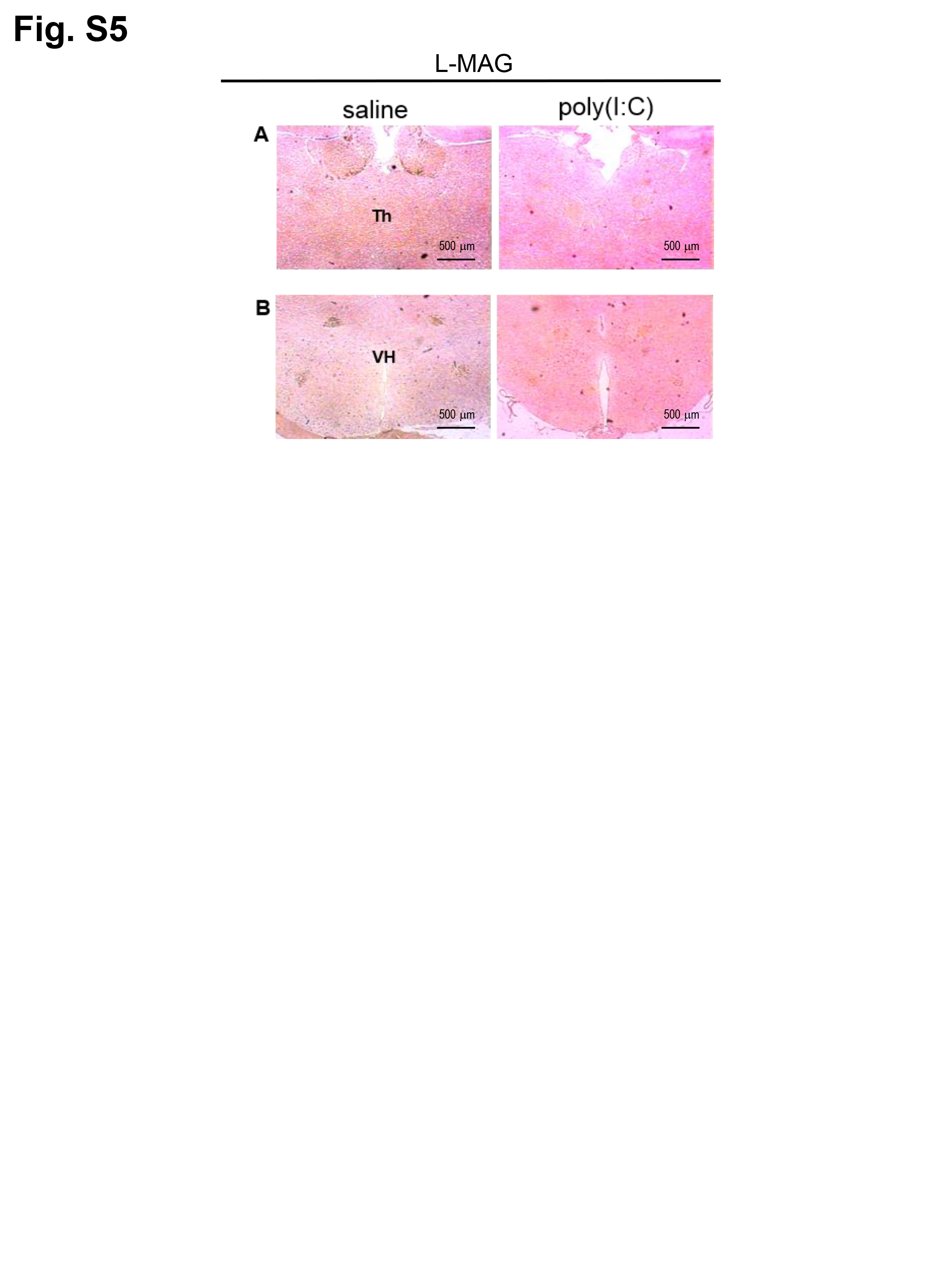

Supplement: FIGURE S5 — In situ hybridization with an L-MAG-specific probe in the thalamus and ventral hypothalamus of adult mouse offspring. L-MAG mRNA was detected as dark blue puncta. Several ROIs in the brain of poly(I:C)- or saline-exposed mice were examined, namely, thalamus (Th; panel A), and ventral hypothalamus (VH; panel B). Results are representative of two independent experiments. Representative images were acquired at 25× magnification. [file Image_5.JPEG]

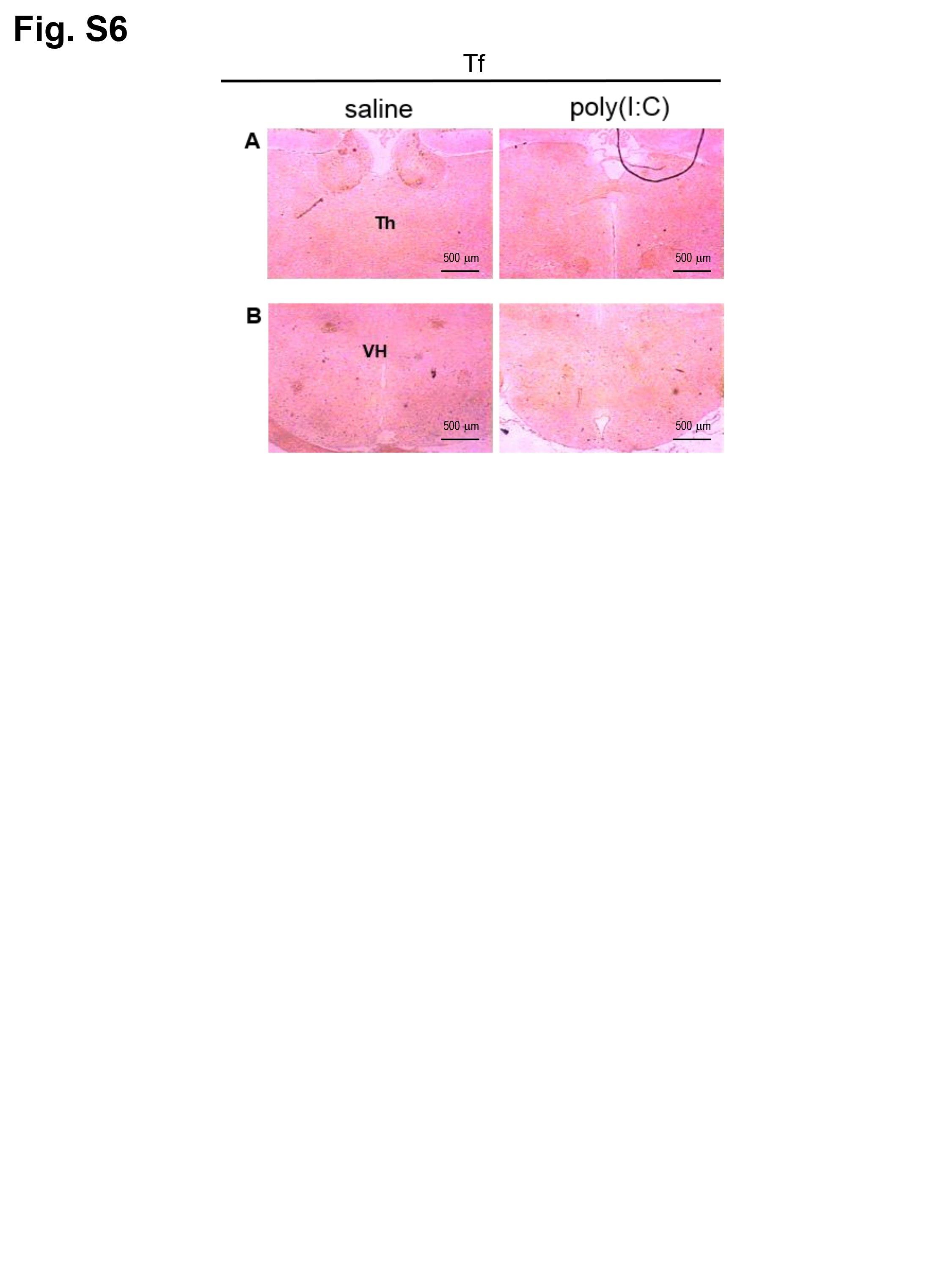

Supplement: FIGURE S6 — In situ hybridization with a Tf-specific probe in the thalamus and ventral hypothalamus of adult mouse offspring. Tf mRNA was detected as dark blue puncta. Several ROIs in the brain of poly(I:C)- or saline-exposed mice were examined, namely, thalamus (Th; panel A), and ventral hypothalamus (VH; panel B). Results are representative of two independent experiments. Representative images were acquired at 25× magnification. [file Image_6.JPEG]
