# Supplementary material for: Poly(I:C) Challenge Alters Brain Expression of Oligodendroglia-Related Genes of Adult Progeny in a Mouse Model of Maternal Immune Activation
Source: Front Mol Neurosci. 2020 Jun 30;13:115. doi: 10.3389/fnmol.2020.00115 (PMC7340146; doi:10.3389/fnmol.2020.00115)
Supplement: TABLE S1 — Primers for generating cDNA templates in molecular cloning. [file Data_Sheet_1.DOCX]

**Table S1.** Primers for generating cDNA templates in molecular cloning

| Gene target | Accession no. | Primer sequences, 5’-3’ |
| --- | --- | --- |
| ***SOX10*** | **NM_011437.1** | Forward primer 5' GTT GGG CTC TTC ACG AGG ACC 3', reverse primer 5' CTG TGG GCA CTG GTG GTG ACA G 3'; nested forward primer 5' ATG GCC GAG GAA CAA GAC CTA 3', nested reverse primer 5' CTA AGG TCG GGA TAG AGT CGT A 3' |
| ***L-MAG*** | **NM_010758** | Forward primer 5' ATG ATA TTC CTC GCC ACC CTG 3', reverse primer 5' TCA CTT GAC TCG GAT TTC TGC 3' |
| ***S-MAG*** | **NM_010758** | Forward primer 5' ATG ATA TTC CTC GCC ACC CTG 3', reverse primer 5' TCA GTG ACA ATC CCG GGT AGA 3' |
| ***transferrin*** | **AF440692.1** | Forward primer 5' ATG AGG CTC ACC GTG GGT GC 3', reverse primer 5' TTA ATG TTT GTG GAA AGT GCA G 3' |
