# Supplementary material for: Poly(I:C) Challenge Alters Brain Expression of Oligodendroglia-Related Genes of Adult Progeny in a Mouse Model of Maternal Immune Activation
Source: Front Mol Neurosci. 2020 Jun 30;13:115. doi: 10.3389/fnmol.2020.00115 (PMC7340146; doi:10.3389/fnmol.2020.00115)
Supplement: TABLE S2 — Primers for gene-specific DIG-labeled probes used in in situ hybridization. [file Data_Sheet_2.DOCX]

**Table S2.** Primers for gene-specific DIG-labeled probes used in *in situ* hybridization

| Gene target | Accession no. | Primer sequences, 5’-3’ |  |
| --- | --- | --- | --- |
| ***SOX10*** | **NM_011437.1** | Forward primer 5' GAA CTG GGC AAG GTC AAG AAG GAA C 3', reverse primer 5' CTC AGC ATT GTG GAG GTG AGG GTA C 3' | |
| ***L-MAG*** | **NM_010758** | Forward primer 5' GAT GCC CTC GAC CAT CTC AGC CTT C 3', reverse primer 5' GGA AAT AGT ATT TGC CTC CCA GCT C 3' | |
| ***Transferrin*** | **AF440692.1** | Forward primer 5' GGC CTG ACT CCG AAC AAC CTG AAG C 3', reverse primer 5' CTG CCC GAG AAG AAA CTG GAC ACA G 3' | |
